# Molecular characterization of carbapenemase and extended spectrum beta-lactamase producing *Acinetobacter baumannii* isolates causing surgical site infections in Ethiopia

**DOI:** 10.1186/s12879-024-09362-5

**Published:** 2024-04-30

**Authors:** Seble Worku, Tamrat Abebe, Berhanu Seyoum, Bikila Alemu, Gebrie Denkayehu, Tamrayehu Seyoum, Dawit Hailu Alemayehu, Alemseged Abdissa, Getachew Tesfaye Beyene, Adane Mihret, Göte Swedberg

**Affiliations:** 1 Department of Microbiology, Immunology and Parasitology, College of Health Sciences, School of Medicine, Addis Ababa University, Addis Ababa, Ethiopia; 2 Department of Medical Laboratory Science, College of Health Sciences, Debre Tabor University, Debre Tabor, Ethiopia; 3 Bacterial and Viral Diseases Research Directorate, Armauer Hansen Research Institute, Addis Ababa, Ethiopia; 4 School of Medical Laboratory Sciences, Jimma University, Jimma, Ethiopia; 5 Department of Surgery, College of Medicine, Debre Tabor University, Debre Tabor, Ethiopia; 6 Department of Medical Biochemistry and Microbiology, Uppsala University, Uppsala, Sweden

**Keywords:** Whole-genome sequencing, Carbapenemase producing, ESBL, *A. baumannii*, SSI, Ethiopia

## Abstract

**Background:**

*Acinetobacter baumannii* is an opportunistic pathogen that can cause a variety of nosocomial infections in humans. This study aimed to molecularly characterize extended-spectrum beta-lactamase (ESBL) producing and carbapenem-resistant *Acinetobacter species* isolated from surgical site infections (SSI).

**Methods:**

A multicentre cross-sectional study was performed among SSI patients at four hospitals located in Northern, Southern, Southwest, and Central parts of Ethiopia. The isolates were identified by microbiological methods and matrix-assisted laser desorption/ionization time-of-flight mass spectrometry (MALDI-TOF MS). Antibiotic susceptibility was determined using disk diffusion. The presence of phenotypic ESBL and carbapenemase production was detected by employing standard microbiological tests, including combined disk diffusion (CDT). ESBL and carbapenem resistance determinants genes were studied by polymerase chain reaction (PCR) and sequencing.

**Results:**

A total of 8.7% *Acinetobacter species* were identified from 493 culture-positive isolates out of 752 SSI wounds. The species identified by MALDI-TOF MS were 88.4% *A. baumannii*, 4.7% *Acinetobacter pittii*, 4.7% *Acinetobacter soli*, and 2.3% *Acinetobacter lactucae.* Of all isolates 93% were positive for ESBL enzymes according to the CDT. Using whole genome sequencing 62.8% of the *A. baumannii* harbored one or more beta-lactamase genes, and 46.5% harbored one or more carbapenemase producing genes. The distribution of beta-lactamases among *Acinetobacter species* by hospitals was 53.8%, 64.3%, 75%, and 75% at JUSH, TASH, DTCSH, and HUCSH respectively. Among ESBL genes, *bla*_CTX−M_ alleles were detected in 21.4% of isolates; of these 83.3% were *bla*_CTX−M−15_. The predominant carbapenemase gene of *bla*_OXA_ type was detected in 24 carbapenem-resistant *A. baumannii* followed by *bla*_NDM_ alleles carried in 12 *A. baumannii* with *bla*_NDM−1_ as the most common.

**Conclusions:**

The frequency of *Acinetobacter species* that produce metallobetalactamases (MBLs) and ESBLs that were found in this study is extremely scary and calls for strict infection prevention and control procedures in health facilities helps to set effective antibiotics stewardship.

## Introduction

Acinetobacter are aerobic, gram-negative coccobacilli bacteria belonging to the Moraxellaceae family and is considered a ubiquitous organism. *Acinetobacter baumannii, A. pittii, A. soli*, and *A. lactucae* are some of many species [1]. Among them, *A. baumannii* has become one of the most common invasive and multidrug resistant (MDR) organisms causing infections in hospital settings [2] and are often associated with poorer clinical outcomes in patients with prolonged hospital stays [3]. Recently *A*. *pittii*, *A. soli* and *A. lactucae* are also considered clinically important [4, 5] and are increasingly recognized as significant causes of infections in hospitals especially with in patients with compromised immune systems [1, 6, 7].

*Acinetobacter species* are associated with a wide range of clinical complications, such as pneumonia, septicaemia, urinary tract infection, and surgical wound infection, and are associated with high mortality, particularly in immune-compromised patients [8], and among long staying patients in hospital settings. Additional risk factors include recent surgery, central vascular catheterization, tracheostomy, mechanical ventilation, enteral feeding, and treatment with third-generation cephalosporin, fluoroquinolone, or carbapenem antibiotics [9].

*A. baumannii* infections pose a global threat to human health and a therapeutic challenge due to emerging and constantly increasing resistance, and carbapenem resistant *A. baumannii* (CRAB) was ranked as the number one priority for antibiotic research and development by WHO 2018 [10]. The development of third-generation cephalosporins was a major development in the fight against multidrug resistant microorganisms. However, due to extensive antibiotic misuse and poor stewardship, resistance to third generation cephalosporins has spread rapidly. The main resistance determinants, extended-spectrum β-lactamases (ESBLs), can make a diverse range of β-lactam antibiotics ineffective, including penicillins, cephalosporins, and monobactams [11]. *Enterobacteriaceae*, *Pseudomonas aeruginosa*, and *A. baumannii* acquire and disseminate ESBL-encoding genes horizontally, mainly through plasmids [12]. Generally, all beta-lactamase variants are classified into four classes, A (serine penicillinases), B (metallo-beta-lactamases), C (cephalosporinases (acinetobacter-derived cephalosporinase or ADC) and D (oxacillinases), which give resistance to penicillins, most β-lactams, cephalosporins and cloxacillin, respectively [13]. The most prevalent ESBL types in the past were TEM, or Temoniera (a Greek name), and SHV, or sulfhydryl variable. However, the most prevalent ESBL type now is CTX-M, which is named after its preferred hydrolytic activity against cefotaxime (CTX, M for Munich), where the CTX-M-15 variant is dominant globally [14].

In addition to third-generation cephalosporin antibiotics, carbapenems are important therapies for serious hospital-acquired infections and the care of patients affected by multidrug-resistant organisms. However, the global emergence of carbapenem-resistant *A. baumannii* has led to limited therapeutic options [15]. Several mechanisms are responsible for the resistance of *A. baumannii* to carbapenems, including reduced outer membrane permeability, penicillin-binding protein alterations, and mostly the production of carbapenemases [11].

*A. baumannii* has been reported in many regions of the world [9, 16]. However, there is a scarcity of data related to the genetic epidemiology of ESBL and carbapenemase producing *A. baumannii* strains in East African countries, including Ethiopia. It is crucial to determine the genetic epidemiology of ESBL and carbapenemase-producing *A. baumannii* strains to guide future antimicrobial resistance control programs. Hence, this study aims to determine the molecular epidemiology of ESBL and carbapenemase-producing *A. baumannii* strains collected among patients investigated for surgical site infections at four Ethiopian Teaching Hospitals, which are placed in the Amhara, Southern nation nationality, Oromia, and central regions of the country. These hospitals serve millions of people in the surrounding catchment areas.

## Materials and methods

**Fig. 1:**
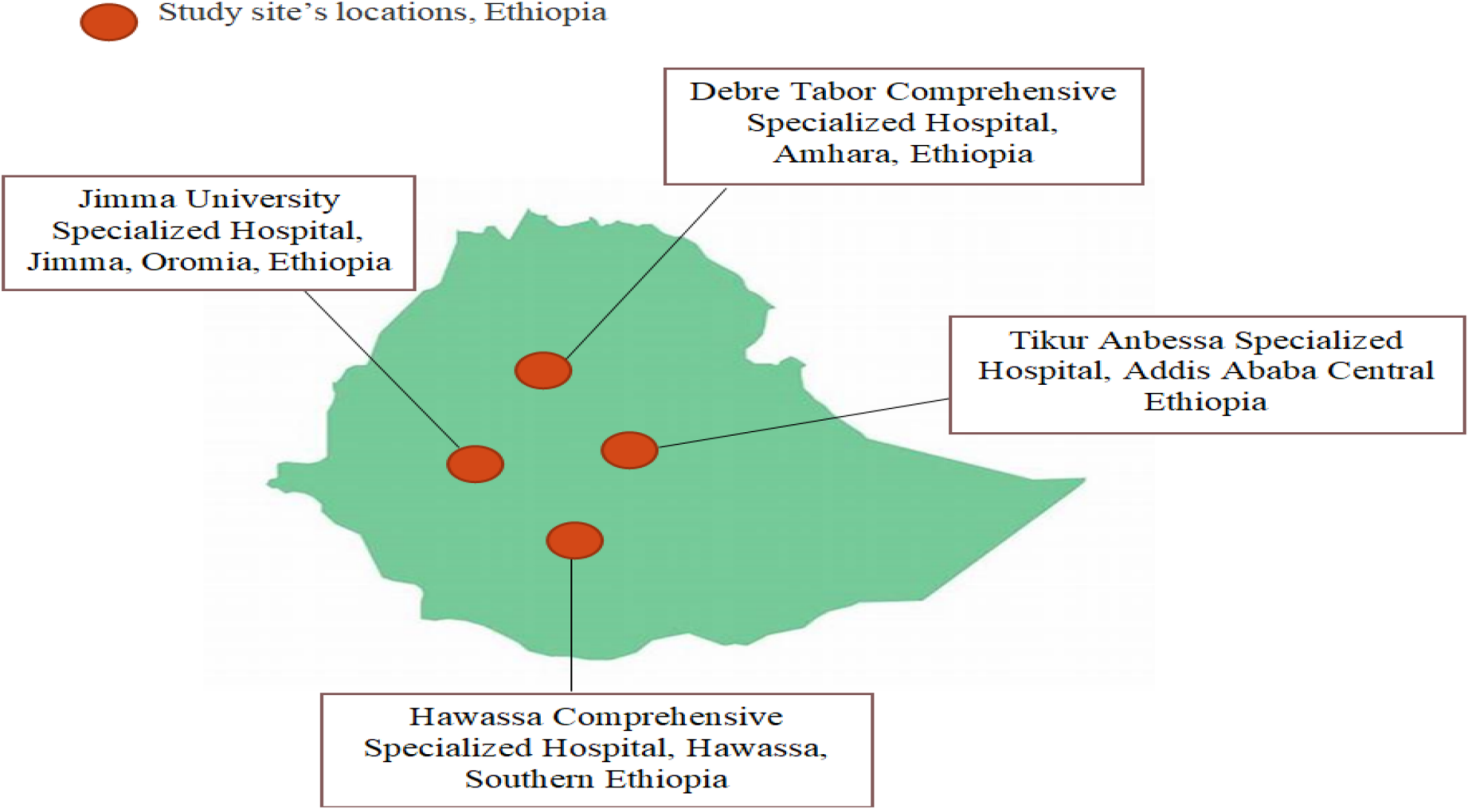
Map of the geographic locations of the four referral hospitals selected for this study

A cross-sectional multicenter study was done at four hospitals in Northern, Central, Southern, and Southwest Ethiopia between July 2020 and August 2021. The purposively selected Hospitals Debre Tabor Comprehensive Specialized Hospital (DTCSH), Hawassa University Comprehensive Specialized Hospital (HUCSH), Jimma University Specialized Hospital (JUSH), and Tikur Anbessa Specialized Hospital (TASH) (Fig. 1) were briefly described in our previous published work by Worku S et al. [17].

The attending physician’s decision was used to identify the eligible SSI patients. After operation, patients were followed by a surgeon to assess the progress of wound healing as part of the routine activity. From all patients whose diagnosis was confirmed as SSI (the infection can be characterized by pain, redness, edema, tenderness, gaping, abscess or purulent discharge, occurrence of fever > 38 °C), from the surgical site within 30 days of the operation for those without implant [18] their socio-demographic and possible risk factor data was gathered. All age groups were included, but patients who had been on antibiotics within the preceding ten days were excluded from the study. A total of 752 clinically diagnosed cases of SSI from different wards in all hospitals were enrolled in the study. Surgical wound swabs or aspirates were collected based on standard operation procedures and processed from all patients. Bacterial identification was performed using a standardized laboratory protocol. At each study site, *A. baumannii* were characterized by their colony characteristics, Gram staining, and conventional biochemical tests. All bacterial strains were stored at − 70 °C and transported to the Armauer Hansen Research Institute (AHRI). Later, all the bacterial isolates were re-identified and confirmed by using Matrix-Assisted Laser Desorption Ionization-Time of Flight Mass Spectrometry (MALDI-TOF MS) [19].

### Antimicrobial susceptibility testing (AST)

The antibiotics susceptibility tests were performed on Muller-Hinton agar (MHA) (Oxoid, UK) by using the Kirby-Bauer disk diffusion technique [19]. Using a sterile wire loop, 3–5 pure colonies were transferred to a tube containing 5 mL of sterile normal saline (0.85% NaCl) and gently mixed. Standard inoculum density was adjusted to 0.5 McFarland units. The excess broth suspension was removed by tapping against the tube wall. The bacterial suspension was swabbed on the MHA surface using a sterile swab, and then antibiotic discs were placed with sterile forceps at least 24 mm apart from one another [20]. All antibiotics disks were OXOID products (Oxoid Ltd, UK), and susceptibility of Gram-negative isolates was tested against: gentamicin (10 µg), amikacin (30 µg), ciprofloxacin (5 µg), ceftazidime (30 µg), cefotaxime (30 µg), ceftriaxone (30 µg), cefepime (30 µg), trimethoprim-sulfamethoxazole (1.25/23.75 µg), ampicillin-sulbactam (10/10µg), meropenem (10 µg), imipenem (10 µg), ertapenem (30 µg) as describes in our previous published work by Worku S et al. [17].

### Screening of ESBL producing strains

Extended-spectrum Beta-lactamase (ESBL) production was confirmed both in the Combination Disk Test (CDT) and by the decreased susceptibility to one of ceftriaxone, ceftazidime or cefotaxime according to the Clinical and Laboratory Standard Institute (CLSI) recommendations [21]. In this test, a disk containing cephalosporin alone (cefotaxime 30 µg or ceftazidime 30 µg) was placed in the opposite direction to a disk containing cephalosporin plus clavulanic acid (20/10µg) with 15 mm distance on Muller-Hinton agar medium.

**Fig. 2:**
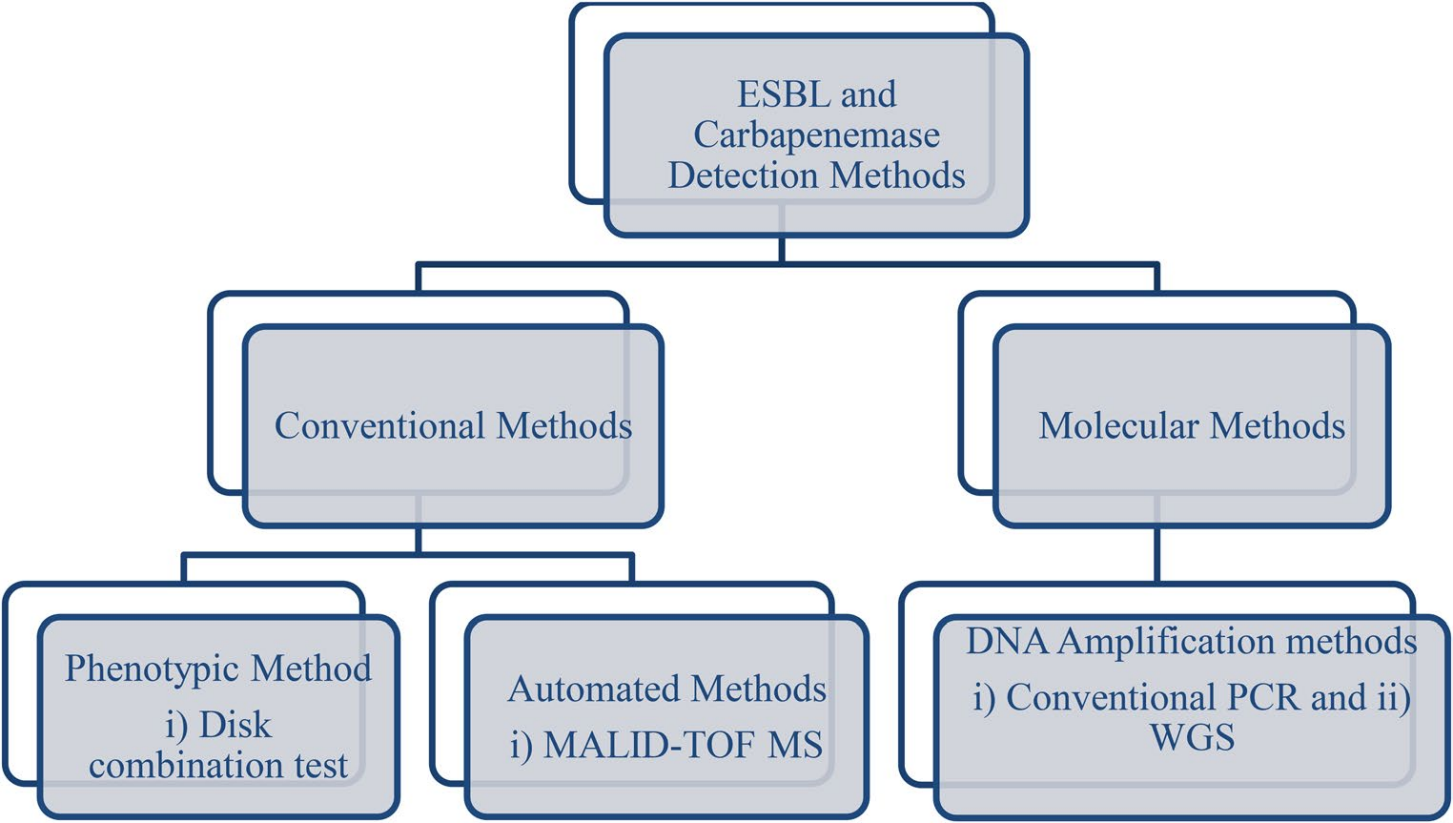
Simple flow chart of extended spectrum beta-lactamase and carbapenemase enzyme detection. ESBL, extended-spectrum beta-lactamase; DNA, deoxyribonucleic acid; PCR, polymerase chain reaction; WGS, whole genome sequencing

The inoculated media were then incubated at 37^o^C for 18–24 h. After incubation, zones of growth inhibition were measured to the nearest mm, and a difference of > 5 mm for a disk containing cephalosporin plus clavulanic acid compared to a disk containing cephalosporin alone was considered positive (see Fig. 2 laboratory workflow chart).

### Detection of ESBL and carbapenemase genes by PCR

All of the positive ESBL (*n* = 40) and carbapenemase (*n* = 40) isolates according to phenotypic assays were further confirmed by PCR and sequencing. The genes investigated in this study were *bla*_TEM_, *bla*_SHV_, and *bla*_CTX−M_. Furthermore, carbapenem-resistant *Acinetobacter species* were tested for *bla*_KPC_ and *bla*_NDM_ like enzymes.

The bacterial DNA was extracted by the boiling lysis method as previously described by El-Badawy et al. [22]. In short, three to five fresh colonies of bacteria were suspended in 100 µl of DNase-free water in a sterile 1.5 ml Eppendorf tube. The bacterial suspension was vortexed for 15 s and placed in a boiling water bath at 94 °C for 10 min to lyse the bacterial cells. The lysed bacterial suspension was centrifuged at maximum speed (13,000 ×g) for 5 min. DNase-free tips were used to transfer the supernatant which contains all of the genomic DNA to a fresh, sterile tube. Nanodrop (Thermo Scientific, US) was used to measure the quality and amount of the extracted DNA, which was then kept at -20^∘^C.

Multiplex PCR was performed to detect *bla*_TEM_, *bla*_SHV_, and *bla*_CTX−M_, as well as *bla*_KPC_ and *bla*_NDM_ carbapenemase genes using specific primers [23] (Table 1). In summary, the PCR was carried out in a QIAGEN Multiplex PCR Master Mix (QIAGEN, Germany) with 0.2µM of each primer, and about 300ng of template DNA in a final volume of 15 µl. Using a thermocycler (Biometra, Germany) for amplification, the following cycling parameters were used: first denaturation at 95 °C for 15 min; subsequently followed by 35 cycles of denaturation at 94 °C for 30 s; annealing at 58 °C for 90 s; extension at 72 °C for 90 s; and a final extension at 72 °C for 10 min. The PCR products were visualized by electrophoresis in 1.5% agarose gel after staining with ethidium bromide. Using a UV trans-illuminator (Bio-Rad, US), the amplicon was visualised and its size was determined using a 100 bp ladder (Promega, US). The PCR products that tested positive were subjected to sequencing. PCR positive samples containing resistance genes were further analysed by whole genome sequencing (WGS).

**Table 1:** Primers used for detection of *bla*_SHV_, *bla*_TEM_, *bla*_CTX−M_, *bla*_KPC,_ and *bla*_NDM_

Target gene	Primer name	CGCCTGTGTATTATCTCCCT	Size bp	References
*bla*_SHV_	F	CGCCTGTGTATTATCTCCCT	293	[24] [25]
	R	CGAGTAGTCCACCAGATCCT		
*bla*_TEM_	F	TTTCGTGTCGCCCTTATTCC	403	
	R	ATCGTTGTCAGAAGTAAGTTG		
*bla*_CTX−M_	F	CGCTGTTGTTAGGAAGTGTG	754	
	R	GGCTGGGTGAAGTAAGTGAC		
*bla*_KPC_	F	CGTCTAGTTCTGCTGTCTTG	798	
	R	CTTGTCATCCTTGTTAGGCG		
*bla*_NDM_	F	GGTTTGGCGATCTGGTTTTC	621	
	R	CGTCTAGTTCTGCTGTCTTG		

### DNA sequencing

The QIAamp DNA Mini Kit (QIAGEN, Hilden, Germany) was used to manually extract DNA in accordance with the manufacturer’s instructions. Briefly, DNA was extracted by taking 2–6 pure colonies that grew on cystine lactose electrolyte deficient agar. After extraction, the concentration of DNA was measured with QubitTM3.0 (Thermo Scientific, Waltham, MA, USA), and kept at -20^o^C until they were submitted for WGS at the Science for Life Laboratory in Solna, Sweden.

From each DNA sample, 20 µL was transferred into a 96-well WGS plate. Sequencing libraries were generated using Nextera XT (Illumina kits) and short-read sequencing was run on Illumina (HiSeq 2500) systems with a 150 bp insert size paired end sequencing protocol at the Science for Life Laboratory. SPAdes (version 3.9) was used for the genome assembly.

With the assembled genomes, the acquired antimicrobial resistance genes were identified using the ResFinder 4.1 web tool at the Center for Genomic Epidemiology http://www.genomicepidemiology.org/ (accessed on August 2023) using a threshold of 90% and 60% coverage. Each WGS run included quality control.

### Quality control

The quality control measures performed throughout the whole process of the laboratory work ensured the validity of the study. Standard operating procedure (SOP) was followed in the collection of all samples. To ensure the accuracy of the data a double data entry method was used. The performance of all prepared media and the potency of the drugs were checked by inoculating control strains, *E. coli* (ATCC 25,922), for each new batch of agar plates. In addition, the sterility of culture media was checked by incubating 5% of the prepared media at 37 °C for 24–48 h. Reagents for Gram-stain and biochemical tests were checked against control strains of *E. coli.* The 0.5 McFarland standard densitometer was used. *Klebsiella pneumoniae* ATCC® 700,603 was used for screening and confirmatory tests for ESBLs (positive). Each MALDI-TOF MS run also included quality control strains using *E. coli* (ATCC® 25,922). During PCR analysis laboratory reference *bla*_KPC_ and *bla*_NDM_ genes were used as positive controls and *E. coli* ATCC1 25,922 as a negative control. Before multiplexing, each pair of primers was verified using monoplex PCR.

### Data analysis

After the data were checked for completeness, missing values and coding of questionnaires, they were entered into Research Electronic Data Capture (RED-Cap) and exported to STATA version 25.0. Frequencies and cross-tabulations were used to summarize descriptive statistics (median, percentages, or frequency). Statistical significance was considered at p-values less than or equal to 0.05.

## Results

### Socio-demographic characteristics of study participants

A total of 493 (65.5%) patients had a positive culture from 752 wound culture tests performed for patients diagnosed with surgical site infection. *Acinetobacter species* were identified from 43 patients. The median age of these patients was 30 years (5 days–70 years) and a total of 21 (48.8%) were females (Table 2). The majority of the bacteria were isolated from surgical wards (21/43) and orthopaedics wards (10/43) of the hospitals as shown in Table 2.

### MALDI-TOF MS identification of *Acinetobacter species*

According to MALDI-TOF MS, a total of 43 *Acinetobacter species* isolates were identified from patients who were admitted to the selected hospitals in Ethiopia. Of the total isolates, 38 (88.4%) were *A. baumannii*, 2 (4.7) *A. pittii* and *A. soli* each, and 1 (2.3%) *A. lactucae*.

**Table 2:** Socio-demographic characteristics of study participants and magnitude of isolated *Acinetobacter species* from SSI Patients at Four Hospitals in Ethiopia, between July 2020 and August 2021

Variables	Characteristics	Total	Beta-lactamase gene		*P* -value
		N (%)	Positive n (%)	Negative n (%)	
Sex	Male	22 (51.2)	19 (86.4)	3 (13.6)	0.63
	Female	21 (48.8)	16 (76.2)	5 (23.8)	
Age (in years)	<= 18	11 (25.6)	8 (72.7)	3 (27.3)	0.37
	19–40	19 (44.2)	15 (78.9)	4 (21.1)	
	41–60	9 (20.9)	8 (88.9)	1 (11.1)	
	≥ 61	4 (9.3)	4 (100)	0 (0)	
Ward	Paediatrics /nicu	6 (14)	5 (83.3)	1(16.7)	0.2
	ICU	2 (4.7)	1 (50)	1 (50)	
	Surgical	24(48.9)	19 (79.2)	5 (20.8)	0.28
	Orthopaedics	11(23.3)	10 (90.9)	1 (9.1)	0.36
Organism isolated	*A.baumannii*	38 (88.4)	32 (84.2)	6 (15.8)	
	*A. pittii*	2 (4.7)	2 (100)	0 (0)	
	*A. soli*	2 (4.7)	0 (0)	2 (100)	
	*A. lactucae*	1 (2.3)	1 (100)	0 (0)	

n: number of *Acinetobacter species*; ICUs: Intensive Care Unit; NICU: neonatal intensive care unit

**Fig. 3:**
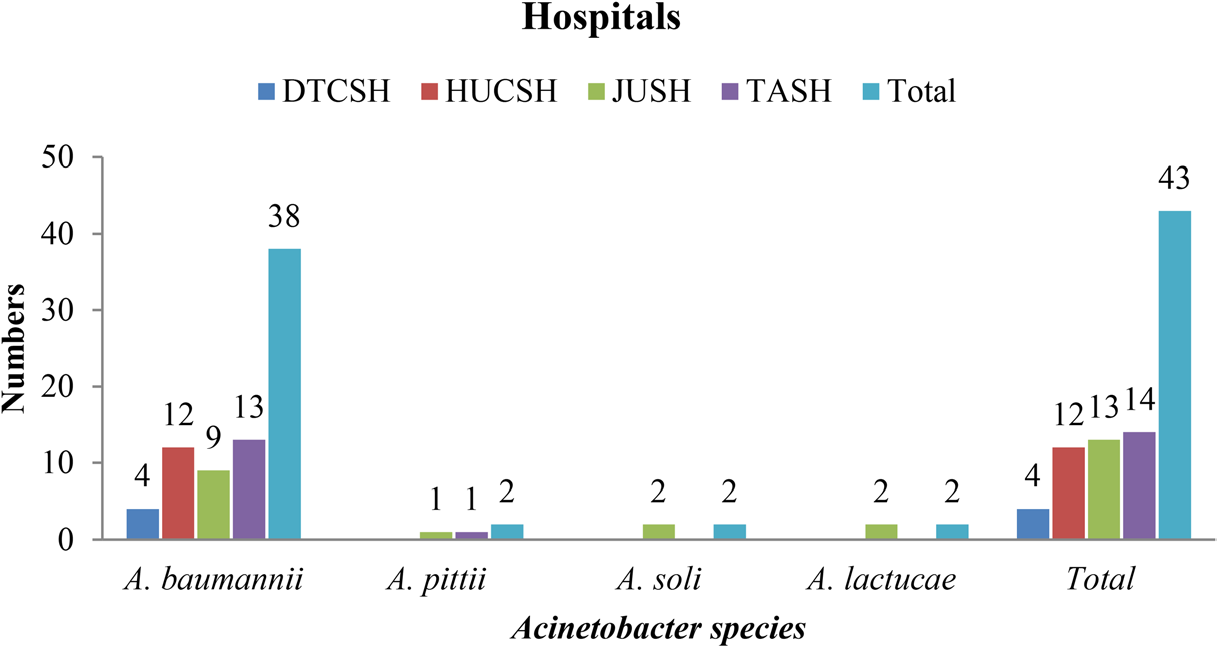
Frequency and distribution of *Acinetobacter species* isolates at each hospital in Ethiopia, between July 2020 and August 2021. DTCSH; Debre Tabor Comprehensive Specialized Hospital; HUCSH: Hawassa University Comprehensive Specialized Hospital: JUTSH; Jimma University Teaching Specialized Hospital; TASH; Tikur Anbessa Specialized Hospital, n: number of *Acinetobacter species*

The majority of the *A. baumannii* isolates were identified from Tikur Anbessa Specialized Hospital and Jimma Specialized Hospital with 14 and 13, respectively, while different species of Acinetobacter were found in Jimma and Tikur Anbessa hospitals (Fig. 3).

### Prevalence of ESBL and carbapenemase producing *Acinetobacter species*

**Fig. 4:**
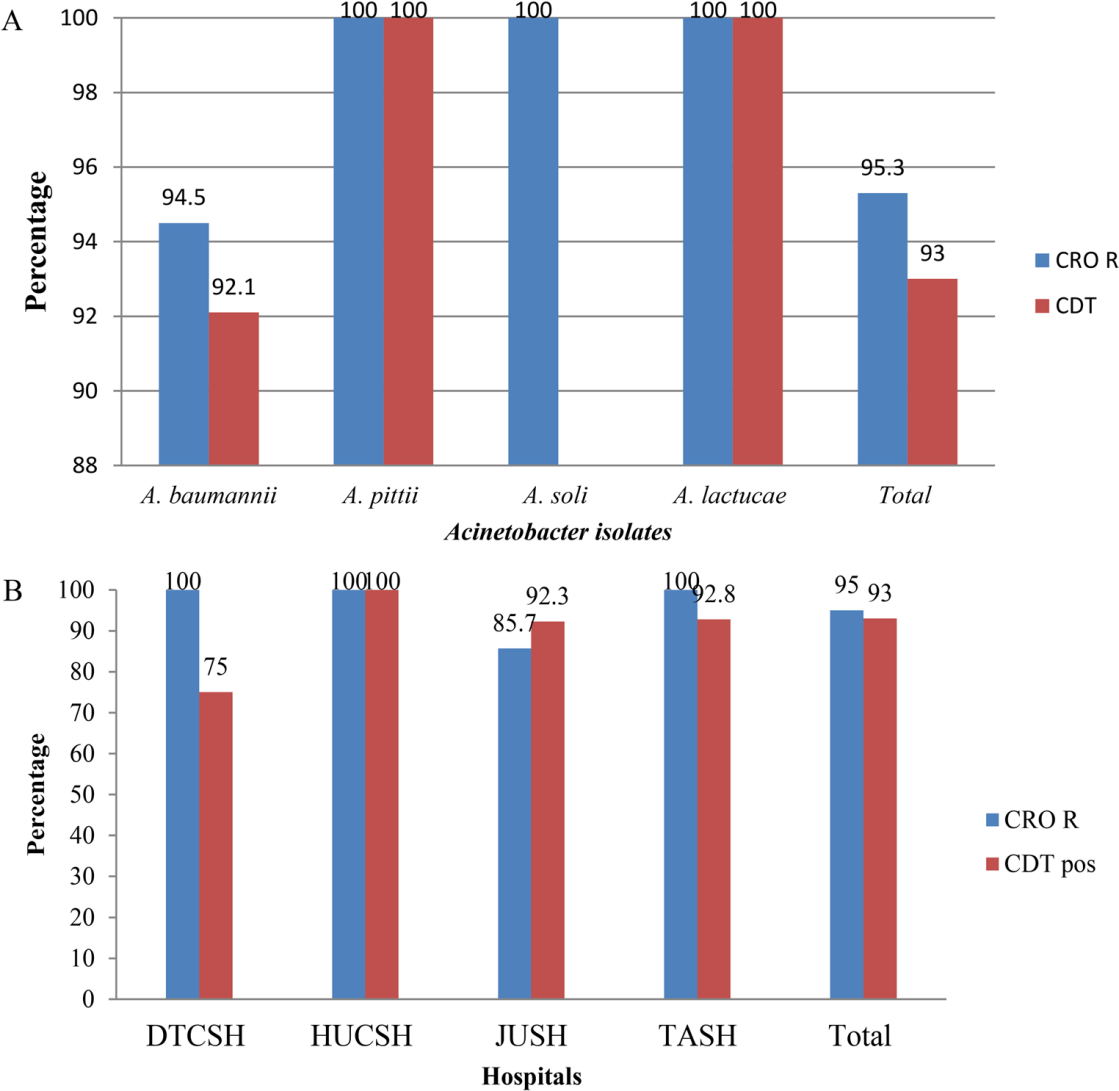
**A** and **B** frequency and distribution of beta-lactamase production among *Acinetobacter species* from patients investigated for surgical site infection at four different hospitals in Ethiopia, between July 2020 and August 2021. DTCSH; Debre Tabor Comprehensive Specialized Hospital; HUCSH: Hawassa University Comprehensive Specialized Hospital: JUTSH; Jimma University Teaching Specialized Hospital; TASH; Tikur Anbessa Specialized Hospital, n: number of bacterial isolates. Abbreviations: CRO R, Ceftriaxone resistance; CDT, combination disc diffusion test

Of the total 43 *Acinetobacter isolates*, 95.3% (41/43) were resistant to ceftriaxone, and 93% (40/43), were confirmed for ESBL production by combined disk-diffusion (CDT), (Fig. 4A).

The highest frequency of ESBL and carbapenemase production was reported from Hawassa Comprehensive Specialized Hospital with a 100% score for ceftriaxone resistance, and CDT positivity. Second was Tikur Anbessa Specialized Hospital with 100%, and 92.8% (Fig. 4B).

### Detection of beta-lactamase genes by whole genome sequencing

**Fig. 5:**
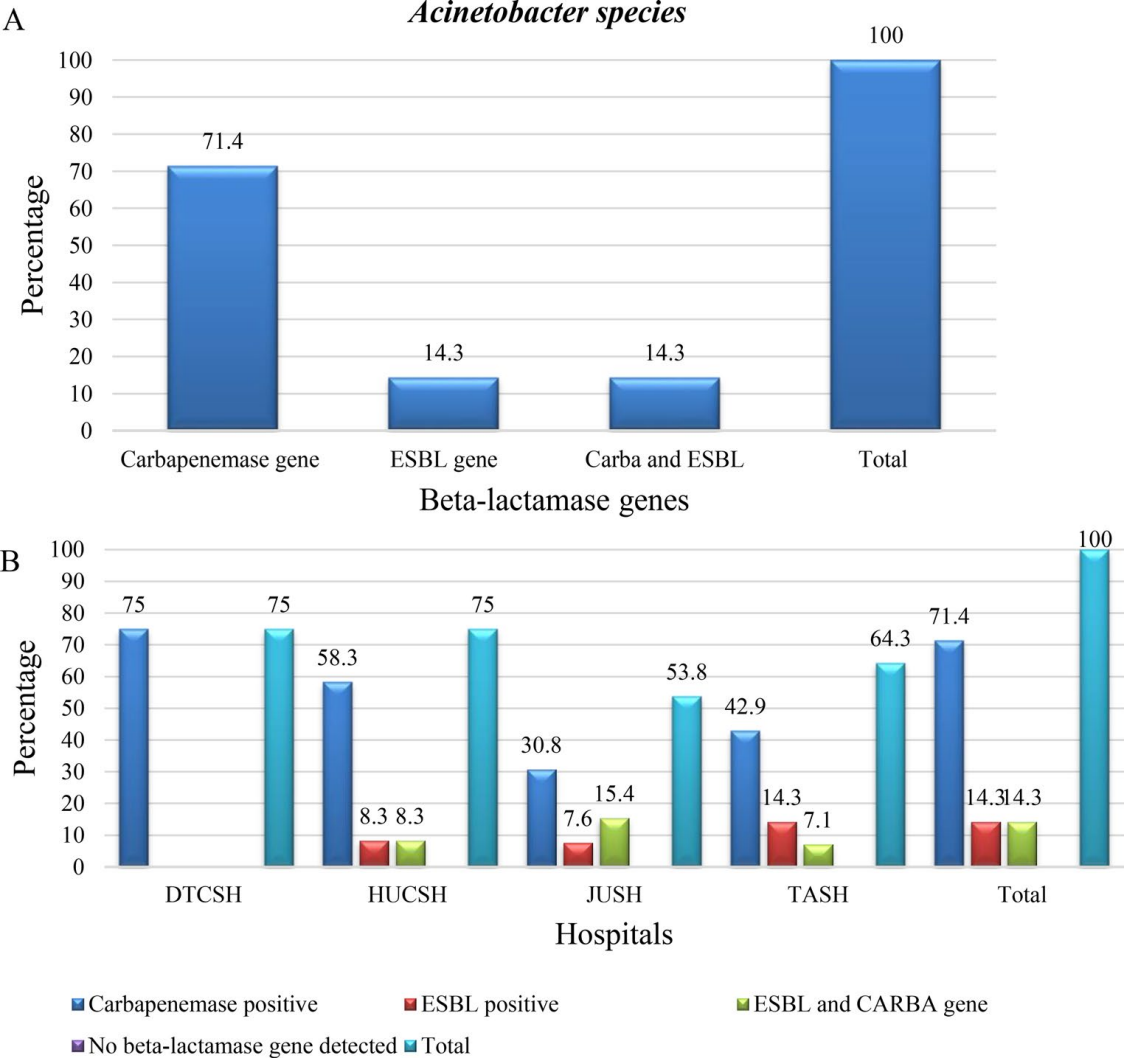
**A** and **B** frequency and distribution of beta-lactamase genes from the total number of *Acinetobacter species* at each Hospital in Ethiopia, between July 2020 and August 2021 **DTCSH**; Debre Tabor Comprehensive Specialized Hospital; **HUCSH**: Hawassa University Comprehensive Specialized Hospital: **JUSH**; Jimma University Teaching Specialized Hospital; **TASH**; Tikur Anbessa Specialized Hospital

One or more beta-lactamase genes were found in 65.1% of *Acinetobacter species*, 71.4% of these had only carbapenemase genes, 14.3% had both carbapenemase genes and ESBL genes, and 14.3% only ESBL genes (Fig. 5A). In *A. baumannii*, 62.8% of the isolates harbored one or more beta-lactamase genes, and 46.5%, 7% and 9.3% of the isolates harbored only carbapenemase genes, only ESBL genes, and carbapenemase and ESBL genes respectively.

Among the isolates at each hospital, the total detection of one or more beta-lactamase genes were 53.8%, 64.3%, 75%, and 75% at JUSH, TASH, DTCSH, and HUCSH respectively (Fig. 5B). In addition, the carbapenemase gene detection at DTCSH, HUCSH, TASH, and JUSH was 75%, 66.7%, 35.7%, and 30.8% (Fig. 5B).

### Carbapenemase and ESBL genes detected by whole genome sequencing

Of 40 *Acinetobacter species* showing ESBL production with combination disk test (CDT), we performed WGS for 28 (27 *A. baumannii* and one *A. lactucae*) isolates, 14.3% (4/28) harbored one or more ESBL genes, 71.4% (20/28) one or more carbapenemase genes, and 100% (28/28) any beta-lactamase gene respectively (Table 3).

Among ESBL genes, one or more *bla*_CTX−M_ alleles were the most commonly identified genes detected from six isolates, five of these were *bla*_CTX−M−15_, and one *bla*_CTX−M−65_ (Table 3). Two *A. baumannii* isolates concurrently harbored *bla*_OXA−1_ with one or more ESBL genes such as *bla*_CTX−M−15_, *bla*_CTX−M−65_, and *bla*_ACT−16_. Among the *Acinetobacter species* isolated at each hospital, the detection of *bla*_CTX−M_ at JUSH, TASH, and HUCSH was 2, 1 and 3 respectively while the *bla*_CTX−M_ allele was not detected at DTCSH. Additionally, *bla*_CTX−M−15_ was carried by one *A. lactucae* concurrently with *bla*_ACT−15_ (Table 3).

**Table 3:** Frequency and distribution of beta-lactamase genes detected among *Acinetobacter species* at each Ethiopian Hospital, between July 2020 and August 2021

Isolates	*ESBL gene (n = 4/28) = 14.3%*	*Carbapenemase gene (n = 20/28) = 71.4%*	*ESBL and CARBA genes (n = 4/28) = 14.3%*	*DTCSH (n = 4)*	*HUCSH (n = 12)*	*JUSH (n = 13)*	*TASH (n = 14)*
A. baumannii (n = 4)		*bla*_*OXA−69*_			3		1
A. lactucae (n = 1)	*bla*_*CTX−M−15*_, *bla*_*ACT−15*_					1	
A. baumannii (n = 2)	*bla*_*TEM−1B*_, *bla*_*CTX−M−15*_				1		1
A. baumannii (n = 1)	*bla*_*TEM−1B*_, *bla*_*CTX−M−15*_						1
A. baumannii (n = 1)			*bla*_*OXA−1*_, *bla*_*CTX−M−65*_			1	
A. baumannii (n = 2)			*bla*_*OXA−69*_, *blaGES*_*− 11*_		1		1
A. baumannii (n = 1)		*bla*_*OXA−23*_, *bla*_*OXA−203*_		1			
A. baumannii (n = 1)		*bla*_*OXA−396*_, *bla*_*OXA−409*_				1	
A. baumannii (n = 3)		*bla*_*OXA−58*_, *blaOXA*_*− 180*_, *blaNDM*_*− 1*_			2		1
A. baumannii (n = 3)		*bla*_*OXA−23*_, *blaOXA*_*− 66*_, *blaNDM*_*− 1*_			1	1	1
A. baumannii (n = 4)		*bla*_*OXA−23*_, *blaOXA*_*− 66*_, *blaNDM*_*− 1*_, *blaADC*_*− 25*_			1	1	2
A. baumannii (n = 1)			*bla*_*OXA−1*,_*bla*_*TEM−1B*_, *bla*_*CTX−M−15*_, *blaACT*_*− 16*_			1	
A. baumannii (n = 2)		*bla*_*OXA−69*_, *bla*_*CARBA−5*_, *bla*_*CARBA−16*_, *bla*_*CARBA−49*_		1		1	
A. baumannii (n = 2)		*bla*_*OXA−69*_, *blaNDM*_*− 1*_, *blaCARBA*_*− 5*_, *bla*_*CARBA−16*_, *blaCARBA*_*− 49*_		1			1
Total (n = 28)				**3 (75%)**	**9 (75%)**	**7 (53.8%)**	**9 (64.3%)**

The *bla*_TEM_ alleles were detected in 4 *A. baumannii*, (Table 3). From these 4 isolates three in addition carried only *bla*_CTX−M−15_ and the remaining one concurrently carried three other ESBL or carbapenemase genes (Table 3).

**Fig. 6:**
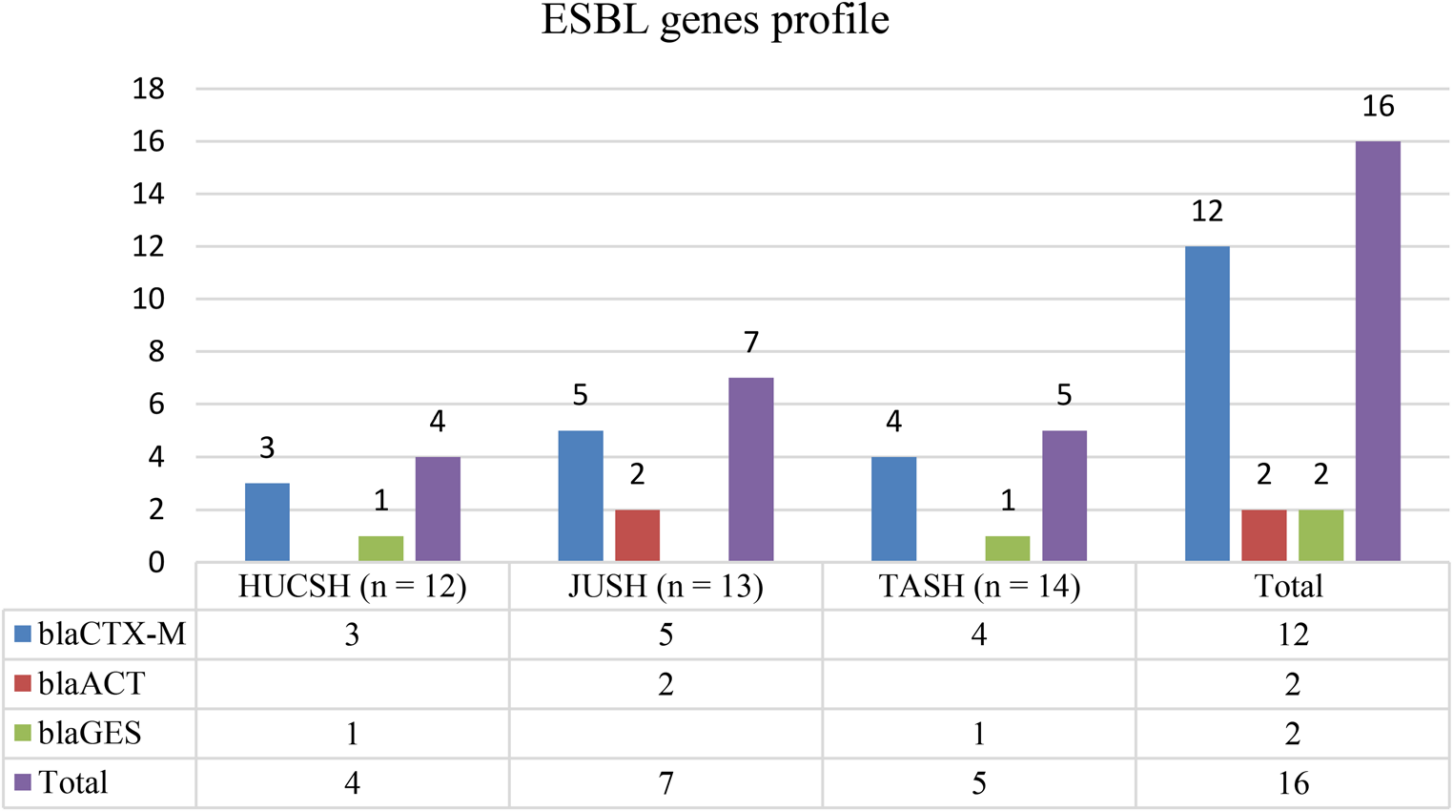
Frequency and distribution of ESBL gene detected at each Hospital in Ethiopia between July 2020 and August 2021. DTCSH; Debre Tabor Comprehensive Specialized Hospital; HUCSH: Hawassa University Comprehensive Specialized Hospital: JUSH; Jimma University Teaching Specialized Hospital; TASH; Tikur Anbessa Specialized Hospital

The predominant carbapenemase genes of *bla*_OXA_-type were detected in 24 (85.7%) of carbapenem resistant *A. baumannii.* Twenty isolates (20/24) carried only one or more carbapenemase genes, four of the isolates carried *bla*_OXA−69_ and 16 isolates showed co-carriage of different *bla*_OXA_-type carbapenemase genes, (Table 3). The second predominant carbapenemase genes were *bla*_NDM_ alleles carried in twelve *A. baumannii* 42.9% (six isolates also carried different alleles of *bla*_OXA_ and four isolates concurrently carried *bla*_ADC−25_). Two isolates concurrently harbored *bla*_NDM_ with *bla*_CARBA−5_, *bla*_CARBA−16_, *bla*_CARBA−49_. (Table 3)

### Detection of *bla*_SHV_, *bla*_TEM_, *bla*_CTX−M_, *bla*_*KPC*_ and *bla*_*NDM*_ with PCR

Of the 40 ESBL and carbapenemase-producing *Acinetobacter species* isolates detected with combined disk-diffusion (CDT), 12 (42.5%) were confirmed for one or more ESBL production genes by multiplex PCR. The *bla*_TEM_, and *bla*_CTX−M_ genes were detected in 10 and 12 isolates respectively, while *bla*_SHV_ was not detected.

**Fig. 7:**
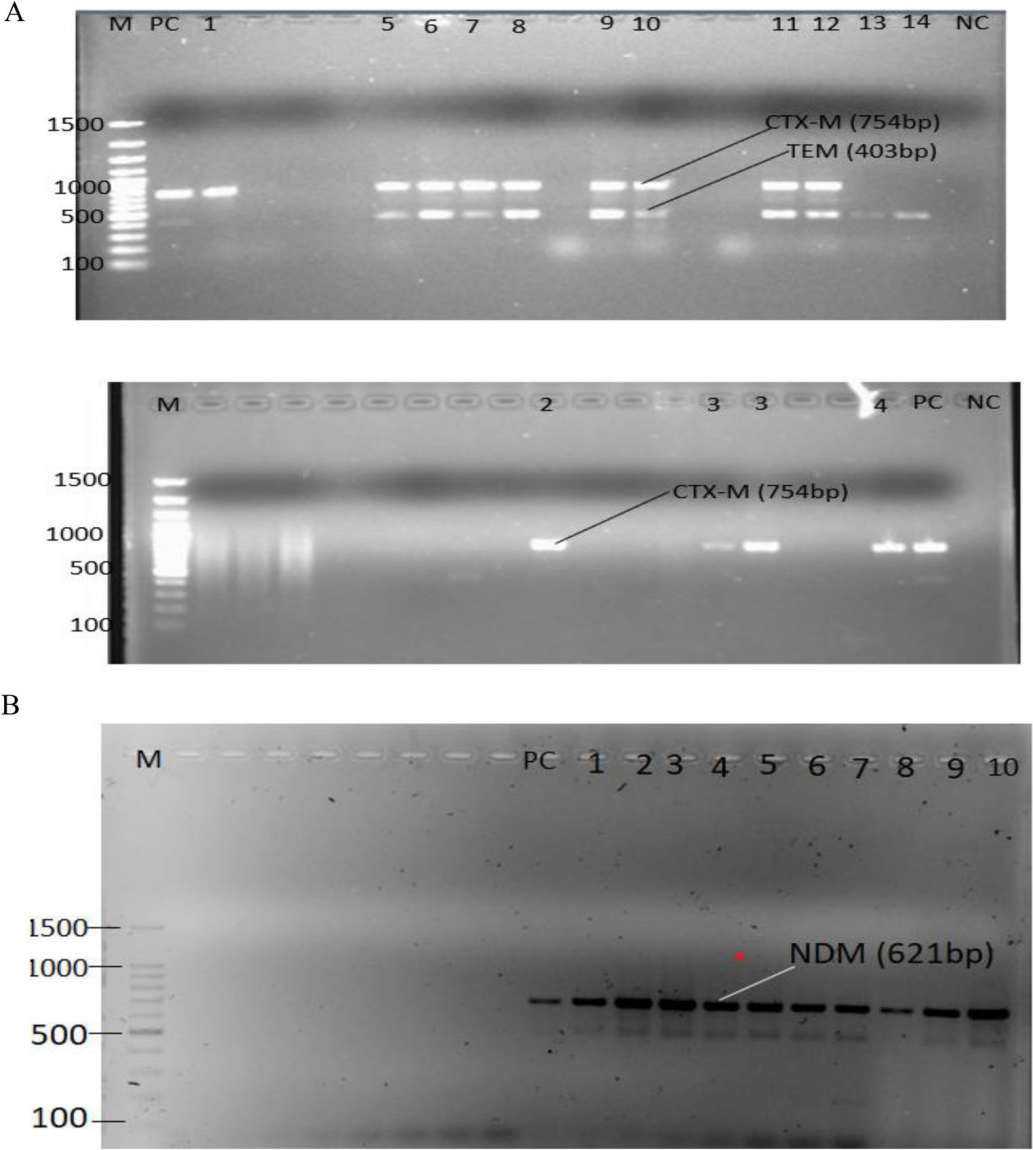
**A** and **B** shows the gel image of the *bla*_CTX−M_ (754 bp), *bla*_TEM_ (403 bp) and *bla*_NDM_ (621 bp) genes. Lane M: 100 bp DNA ladder, PC: Positive control, Lanes 1–14: Acinetobacter isolates, NC: Negative control

Of the 40 MDR and ESBL-producing *Acinetobacter species* isolates according to our previous published work Worku S et al. [17]. , only 12 (30%) *A. baumannii* isolates were carbapenemase gene positive in the multiplex PCR. The *bla*_*NDM*_ gene was detected in 12 isolates (only one isolate carried *bla*_CTX−M_ and *bla*_TEM−1_ genes) while the *bla*_*KPC*_ gene was not detected. Figure 7 shows the gel image of the *bla*_CTX−M_ (754 bp), *bla*_TEM_ (403 bp) and *bla*_NDM_ (621 bp) genes.

## Discussion

This study examined the prevalence of novel beta-lactamase-mediated resistance mechanisms in cephalosporin- or carbapenem-resistant isolates among patients investigated for surgical site infection at four referral hospitals located in the Amhara region, Addis Ababa, southern region and Oromia region of Ethiopia. In our findings the emergence of various coexisting ESBL and carbapenemase-resistance-producing genes in Acinetobacter is alarming and challenging, especially for medical professionals. Those genes pose a major threat globally and may significantly limit the treatment options in hospital settings. Similarly *A. baumannii* has been reported worldwide and has become a significant health problem [26]. Especially carbapenem-resistant *Acinetobacter species*, a critical priority for the World Health Organization, urgently require new antibiotics [10].

In this study, 95.3% *Acinetobacter species* were resistant to ceftazidime. This finding was comparable with the previous study conducted in India [27]. Additionally, in this study, the phenotypic ESBL production by combined disk diffusion test was 93%. This result is higher than studies conducted by Kaur 27.5% [27] and Chaudhry 46.0% [28].

In the present study, the most common ESBL genes detected were *bla*_CTX−M_ from 6 isolates 21.4%. This data is comparable with the previous study reported from Saudi Arabia which was 20% [29] and lower than study reported in Nigeria (25%) [30]. On the other hand in our study, the *bla*_TEM_ gene was detected in 14.3% of the isolates which is lower than the study conducted in Saudi Arabia 70% [29].

The present findings revealed that the *bla*_SHV_ was not detected in any of the isolates. The result is similar to studies conducted in Iran [31] and Algeria [32]. However, the *bla*_SHV_ gene was common in *A. baumannii* isolated in Iraq 25% [33]. These variations could be due to different antibiotic use, and difference in study settings [34].

In this finding 43 (85.7%) isolates were carbapenem-resistant, which is similar with a study conducted in Pakistan (89.1%) [35]. In *A. baumannii*, carbapenem resistance is frequently linked to the existence of metallo-β-lactamases (MBL) such as *bla*_NDM−1_ elsewhere in the world [36, 37]. Similarly in our study, the *bla*_NDM−1_ gene was detected in 25.6% (11/43) *A. baumannii* isolates. In addition, our study is similar to the previous studies conducted in Libya [38], and Algeria [39]. On the other hand, two of the *bla*_*NDM−1*_ genes were detected in *A. baumannii* from Jimma Hospital. This result was comparable with the first *bla*_NDM_ reported from Jimma Hospital [40]. The predominant carbapenemase gene was *bla*_OXA_ type at 58.1%, (mainly *bla*_OXA−23_, and *bla*_OXA−69_) followed by metallo-β-lactamase *bla*_NDM_ (27.5%), genes. This was comparable with the previous study conducted in Ethiopia [41].

More than one ESBL resistance gene in a single isolate increase the difficulty of treating with beta-lactam antibiotic drugs [14]. In this study, the co-existence of two different ESBL genes was frequently detected in a single isolate, similar to a study conducted in Saudi Arabia [29]. The present study revealed that the co-existence of two or more carbapenemase encoding genes in a single isolate was 80% (20/25). This finding is higher than the study conducted in Jimma, Ethiopia [41] and comparable with the study conducted in Tunisia 82% [42].

Many isolates also carried one or more other carbapenemase genes together with ESBL genes showing dissemination of multidrug-resistant (MDR) *A. baumannii* in Teaching and referral Hospitals in Ethiopia. The finding gives an alarming sign towards *A. baumannii* carrying both metallo-beta-lactamases and ESBL production genes conferring resistance to carbapenems and cephalosporins respectively. This combination of resistance genes can limit therapeutic options [26]. Early detection, strict adherence to infection control procedures and antimicrobial policy are the best lines of defence against *A. baumannii*.

Moreover, the widespread distribution of NDM-1 metallo-β-lactamase necessitates special consideration because the enzyme confers resistance to a wide spectrum of beta-lactam antibiotics on the bacteria, and their genetic makeup exhibits remarkable adaptability and mobility. Serious public health problems could arise from the spread of such plasmids across many clinically significant bacterial species, especially GNB *A. baumannii*, in hospital settings [43].

Likewise, the *bla*_OXA−23_ gene is one of the common causes of resistance conferring high level of resistance and was detected in 8 isolates (18.6%). This figure is higher than the study conducted in China which was 4.5% but lower than studies conducted in Libya with 29 strains (80.6%) [38] and Pakistan (97.8%) [35]. The prevalence of this gene may vary in the geographic area and the type of *Acinetobacter species.*

## Conclusions

Our results suggest the existence of different species of Acinetobacter including *A. baumannii, A. pittii, A. soli* and *A. lactucae* in th**e** hospital settings. In the present study carbapenemase-producing genes were detected in 85.7% of *A. baumannii*.

The present finding showed ESBL-producing genes among the isolates, with *bla*_CTX−M_ variants being the most prevalent type and *bla*_CTX−M−15_ gene the predominant variant. In addition, the co-existence of two different ESBL genes was frequently detected in a single bacterial pathogen.

In addition, co-existence of two or more different carbapenemase genes was frequently detected in a single bacterial pathogen with *bla*_OXA_ variants being the most prevalent type and with *bla*_OXA−23,_ and *bla*_OXA−69_ as the predominant variants followed by *bla*_OXA−66_. The second predominant carbapenemase gene was *bla*_NDM−1_.

The emergence of various ESBL and carbapenemase-resistance-producing coexisting genes in Acinetobacter is alarming and challenging, especially for medical professionals. Those genes pose a major threat globally and may significantly limit the treatment options in hospital settings.

The prevalence of ESBLs and MBLs-producing *A. baumannii* strains detected in this study is a major concern and highlights the need for infection prevention and control measures.

## Limitation of the study

The number of *Acinetobacter isolates* was small and may not be representative for the presence of Acinetobacter in the community.

## Data Availability

The data sets generated during and/or analysed during the current study are available from the corresponding authors on reasonable request.
